# The associations between depressive symptoms, functional impairment, and quality of life, in patients with major depression: undirected and Bayesian network analyses

**DOI:** 10.1017/S0033291722003385

**Published:** 2023-10

**Authors:** Jia Zhou, Jingjing Zhou, Lei Feng, Yuan Feng, Le Xiao, Xu Chen, Jian Yang, Gang Wang

**Affiliations:** 1 The National Clinical Research Center for Mental Disorders & Beijing Key Laboratory of Mental Disorders, Beijing Anding Hospital, Capital Medical University, Beijing, China; 2 Advanced Innovation Center for Human Brain Protection, Capital Medical University, Beijing, China

**Keywords:** Functioning disability, life satisfaction, major depressive disorder, network analysis

## Abstract

**Background:**

Depressive symptoms, functional impairment, and decreased quality of life (QOL) are three important domains of major depressive disorder (MDD). However, the possible causal relationship between these factors has yet to be elucidated. Moreover, it is not known whether certain symptoms of MDD are more impairing than others. The network approach is a promising solution to these shortfalls.

**Methods:**

The baseline data of a multicenter prospective project conducted in 11 governances of China were analyzed. In total, 1385 patients with MDD were included. Depressive symptoms, functioning disability, and QOL were evaluated by the 17-item Hamilton Depression Rating Scale (HAMD-17), the Sheehan Disability Scale (SDS), and the Quality of Life Enjoyment and Satisfaction Questionnaire-Short Form (Q-LES-Q-SF). The network was estimated through the graphical Least Absolute Shrinkage and Selection Operator (LASSO) technique in combination with the directed acyclic graph.

**Results:**

Three centrality metrics of the graphical LASSO showed that social life dysfunction, QOL, and late insomnia exhibited the highest strength centrality. The network accuracy and stability were estimated to be robust and stable. The Bayesian network indicated that some depressive symptoms were directly associated with QOL, while other depressive symptoms showed an indirect association with QOL mediated by impaired function. Depressed mood was positioned at the highest level in the model and predicted the activation of functional impairment and anxiety.

**Conclusions:**

Functional disability mediated the relationship between depressive symptoms and QOL. Family functionality and suicidal symptoms were directly related to QOL. Depressed mood played the predominant role in activating both anxiety symptom and functional impairment.

## Introduction

In 2017, the World Health Organization reported that major depressive disorder (MDD) was the second most prevalent disorder globally, affecting more than 300 million people worldwide (WHO, 2017). According to the Diagnostic and Statistical Manual of Mental Disorders, 5th Edition (DSM-5) criteria for MDD, at least five of the following symptoms (i.e. persistently low or depressed mood, anhedonia or decreased interest in pleasurable activities, feelings of guilt or worthlessness, lack of energy, poor concentration, appetite changes, psychomotor retardation or agitation, sleep disturbances, or suicidal thoughts) need to be present during the same 2-week period. And of them, at least one must be either depressed mood or loss of interest (or pleasure) (Bains & Abdijadid, 2022). Even though these symptoms are still defining features of MDD, there is a growing consensus that the scope of evaluation should include broader dimensions (Zhao et al., 2017), for instance, functioning (e.g. social and occupational functioning impairments that disrupt work, school, leisure, family life activities, and family responsibilities) (Sheehan, Nakagome, Asami, Pappadopulos, & Boucher, 2017) and quality of life (QOL) (e.g. subjective evaluation covering physical, mental, and social domains) (Rapaport, Clary, Fayyad, & Endicott, 2005; Yang et al., 2021).

In fact, depressive symptoms, functional impairment, and QOL are significantly inter-correlated (Greer, Kurian, & Trivedi, 2010; Prelipceanu, Purnichi, Marinescu, & Matei, 2015). For example, some cohort studies suggested that depression may lead to functional disability, a type of functional impairment (De Ronchi et al., 2005; Esposito et al., 2021; Simning & Seplaki, 2020), while some other studies indicated that functional disability may increase the risk for subsequent depression (Lyness, Caine, Conwell, King, & Cox, 1993; Noh, Kwon, Park, Oh, & Kim, 2016). In some studies, QOL predicted increased work disability (Koivumaa-Honkanen et al., 2004), whereas some other studies indicated that functional impairment could be an important determinant of QOL, since it occurs early in the course of the disorder and has a significant impact on daily living (Domínguez-Martínez, Kwapil, & Barrantes-Vidal, 2015). It has also been proposed that the experience of disability partially mediates the relationship between symptoms and QOL (Hambrick, Turk, Heimberg, Schneier, & Liebowitz, 2003). However, the causal relationship between depressive symptoms, disability, and QOL has not been elucidated.

Moreover, individual depressive symptoms may be differentially associated with functional impairments and QOL (Fried et al., 2020; Gollan et al., 2012). There is a growing need to identify the domain-specific relationships between depressive symptoms, dysfunction, and QOL that might aid in the identification of treatment targets for specific symptoms to improve functioning and QOL. The STAR*D study found that a sad mood and concentration problems had the highest unique associations with dysfunction, whereas hypersomnia, mid-nocturnal insomnia, and weight problems exhibited little contributions to functional impairment (Fried & Nesse, 2014). Another study found that motivational deficits (reduced interest and drive to initiate and maintain goal-directed activities) demonstrated the most robust impact on functional outcomes (Fervaha, Foussias, Takeuchi, Agid, & Remington, 2016). In terms of QOL, individuals who are depressed may report poorer QOL caused by distress associated with core depressive symptoms, such as negative thinking and low mood (Tang, Thomas, & Larkin, 2019). Wang et al. reported that cognitive symptoms were particularly associated with QOL (Wang, Tan, Ren, & Hammer-Helmich, 2020). Self-stigma level has also been reported to have a negative impact on QOL in patients with MDD (Holubova et al., 2016). However, it remains unclear whether certain symptoms are more impairing than others, and if so, what the magnitude of these differences might be.

The network approach is a promising solution as it brings a novel network perspective relating to psychopathology. It would provide unique information on dynamic and causal relationships among depressive symptoms (Borsboom, 2017) using a developing methodology (Smith et al., 2019). The network theory posits that different symptoms are not equivalent or interchangeable (Fried, 2017). Recent applications of Bayesian approaches based on the directed acyclic graph (DAG) helped researchers to find the critical pathways of activation among symptoms (McNally, 2016). And causality could be inferred by examining the dominant pathways of activation that lead to social dysfunction and a reduction in life satisfaction (Moffa et al., 2017). These methods may propose new directions to improve treatment and may promote a more appropriate allocation of scarce medical resources (O'Driscoll et al., 2021).

Based on this background, we hypothesized that there are potential sequential relationships among dimensions of depressive symptoms, functional impairment, and QOL. In addition, we hypothesized that clinical symptoms and dysfunction have domain-specific associations with QOL. These hypotheses were tested on a large sample of patients with MDD using undirected network analyses and DAG.

## Methods

### Participants and study design

This was a multicenter and prospective project featuring two studies with consistent criteria for enrollment and intervention in the acute phase. The project was conducted at 12 sites in 11 governances of China, including Beijing, Guangdong, Xi'an, Hebei, Shaanxi, Yunnan, Heilongjiang, Jiangsu, Shanghai, Shanxi, Sichuan, and Shenzhen covering the east, south, north, and west of China. These 12 hospitals are located in densely populated cities, of which eight were general hospitals and four were psychiatric hospitals. The patients were enrolled from 9th November 2016 to 30th December 2020. The protocol of the study was approved by the Institutional Review Board (IRB) of Beijing Anding Hospital and an independent medical ethics committee board for all sites. Written informed consent was obtained from all participants. The study protocol was registered and several biomarker studies using single-center biological data have been published (Yang et al., 2020, 2022; Zhou et al., 2021)

The participants were aged 18–65 years and had been diagnosed with MDD according to the Diagnostic and Statistical Manual of Mental Disorders, Fourth Edition (DSM-IV) criteria. A total score of the 17-item Hamilton Rating Scale for Depression (HAMD-17) ⩾ 14, and a total score on the 16-item Quick Inventory of Depressive Symptoms-Self-Report (QIDS-SR16) ⩾ 11 were required. Patients with serious physical diseases and female patients who were pregnant were excluded. Patients at high risk of suicidal ideation (determined by a HAMD-17 item-3 score >2 points) were not included. Patients were excluded if they had used antidepressants continuously for more than 7 days in the most recent 14 days. A total of 1487 patients were enrolled; 102 cases were excluded due to incomplete questionnaires.

The interviews were conducted by trained interviewers who received uniform training before the project was launched. The training included practice scoring with feedback from an expert group and one-on-one discussion with raters who rated differently from others. Inter-rater reliability (kappa values for categorical measures, intra-class correlation for continuous variables) was >0.8 for all measurements. The study was supervised throughout its duration, and there were unscheduled spot checks to ensure good inter-rater reliability.

### Measures

#### 17-item Hamilton Rating Scale for Depression (HAMD-17)

The HAMD-17 was translated into Chinese and validated in patients with depression in 1988 (YP et al., 1988). It is the most commonly used scale to measure symptomatology and illness severity for MDD and has been used for almost 30 years (Lin et al., 2018). The HAMD-17 consists of 17 questions with Likert scale responses of either 0–4 or 0–2; the total scores can range from 0 to 52. Internal consistency (Cronbach's *α*) for the HAMD-17 was 0.829 and 0.615 in a previous study (Ma et al., 2021) and the current baseline sample, respectively. The Cronbach's *α* for the week 4 HAMD-17 is 0.834.

#### Sheehan Disability Scale (SDS)

As a global self-rated questionnaire on functional disability, the SDS was used to evaluate the degree of dysfunction relating to family life/home responsibility, social life/leisure activities, and work/school (Sheehan et al., 2016). These three items were graded according to a visual analogue scale ranging from 0 to 10; this can also be summed as the total SDS score. Internal consistency (Cronbach's *α*) for the SDS was 0.940 and 0.819 in a previous study (Leu et al., 2015) and the current sample, respectively.

#### Quality of Life Enjoyment and Satisfaction Questionnaire – Short Form (Q-LES-Q-SF)

The Q-LES-Q-SF is a self-administered questionnaire that is used to measure health-related QOL covering various domains of well-being. The Q-LES-Q-SF has been a stand-alone measure used extensively in patients with MDD (Zhao et al., 2017). This questionnaire features 16 items scored on a five-point Likert scale ranging from 1 (very poor) to 5 (very good). The total score was computed by summating the scores on the first 14 items. Items 15 and 16 collate information related to satisfaction with the use of medication and overall life (Katila et al., 2013). This scale has been proven to be a valid and reliable assessment tool for measuring QOL in patients with major psychiatric disorders (Ritsner, Kurs, Gibel, Ratner, & Endicott, 2005). Internal consistency (Cronbach's *α*) for the Q-LES-Q-SF was 0.87 and 0.842 in a previous study (Lee et al., 2014) and the current sample, respectively.

### Data analyses

#### Network analysis

First, the R-package graph package (Epskamp, Cramer, Waldorp, Schmittmann, & Borsboom, 2012) was used to construct a partial correlation network (Epskamp, Borsboom, & Fried, 2018) of (i) 17 symptoms of MDD, (ii) three functional disability items, and (iii) QOL (online Supplementary Table S1) in patients with MDD, using the Least Absolute Shrinkage and Selection Operator (LASSO) (Friedman, Hastie, & Tibshirani, 2008). Within the graphical network, each node depicts a symptom, and varying thicknesses of the edges represent the magnitude of regularized partial correlations between two individual symptoms. The stronger the connection is, the stronger, thicker, and more saturated an edge is. Blue edges reflect positive associations while yellow edges reflect negative associations.

Second, we also calculated centrality measures, such as strength, betweenness, and closeness, for all nodes. These metrics examined the relative importance and influence of symptoms (Boccaletti, Latora, Moreno, Chavez, & Hwang, 2006; Opsahl, Agneessens, & Skvoretz, 2010). Node strength was calculated by summating all the edge weights attached to a given node, and it was a common and stable central metric. Betweenness was measured with the number of shortest paths that passed through the node of interest. Closeness referred to the inverse of the sum distance of a node to all other nodes. Highly central symptoms scored the highest on measures of strength centrality, as these strongly affect other symptoms in the network (McNally, 2021). The activation of a highly central symptom increases the likelihood of activation spreading to a large number of other symptoms.

Third, network accuracy and stability were estimated using R-package bootnet (Epskamp et al., 2018); this involved: (a) estimation of the bootstrapped confidence intervals of edge-weights by calculating their 95% confidence intervals (CIs) derived from a non-parametric bootstrap procedure (*n* = 1000); (b) evaluation of the stability of centrality indices from a case-drop bootstrap procedure, in which centrality indices were repeatedly calculated from subsets of data with an increasing proportion of cases subtracted. The correlation stability (CS) coefficient was used to quantify the stability of centrality indices and represents the maximum proportion of cases that can be dropped to retain a correlation of 0.7 in at least 95% of the sample. The CS coefficient should not be below 0.25 and preferably above 0.5; (c) bootstrapped difference tests (*α* = 0.05) for edge-weights and node strength using 1000 bootstrap samples. Gray boxes indicate nodes that do not significantly differ from one another. Black boxes indicate a significant difference.

Fourth, a Bayesian network, derived from the principles of causal reasoning, is defined as the combination of a DAG and a probability distribution (Briganti, Scutari, & McNally, 2022). The DAG encodes conditional independence relationships and characterizes the joint probability distribution of the variables by using graphical separation. The logic behind the use of DAG to depict hypothetical causal structures is set out in detail by Pearl (Pearl, 2000). While causality can never be proved using observational data only, DAG can still provide admissible evidence of causal relationships. It gives information about both the magnitude and direction of the connections between symptoms. DAG has been increasingly used in the psychological context (Moffa et al., 2017). The R-package bnlearn was used in this study. It uses a hill-climbing algorithm (McNally, 2016), as described recently (Amore et al., 2020; McNally, Mair, Mugno, & Riemann, 2017). To ensure the stability of the resultant network, we conducted bootstrapping (*n* = 1000 iterations) and calculated the goodness-of-fit index (i.e. Bayesian Information Criteria, BIC) for each sample (Scutari & Nagarajan, 2013). If those edges displayed the same direction in at least 85% of these networks, then we retained the edge in the resultant network (França, Gordon, Samra, Rodolpho Duarte, & Jacinto, 2020; Moffa et al., 2017). All analyses were carried out in R 3.6.3 (R Foundation for Statistical Computing, Vienna, Austria).

Fifth, we also performed online Supplementary analysis. There was a large gender disparity in participants (only 32% were male), depression was more common in females, and some of the factors examined (e.g. family factors) differed by gender. Given these factors, it was important to examine whether the findings differ in males and females. We compared the gender difference in the network using the recently developed test, the Network Comparison Test (NCT), which allows researchers to test differences in global network strength (i.e. the overall level of connectivity of the network) and global network structure (i.e. the overall structure of the network) between two networks (Borkulo et al., 2017). The Q-LES-Q-SF has 16 items and includes the same content as the general activities section of the longer version (Lee et al., 2014). No criteria that can help to categorize the items into dimensions were found. If all individual items of the Q-LES-Q-SF were included as nodes in the network analyses, it would be difficult to interpret. Therefore, only the total score of the Q-LES-Q-SF was included. The result of the undirected network analysis is also added in the online Supplementary material, in which items of the Q-LES-Q-SF were included (online Supplementary Fig. S7). In addition, we used the follow-up data at week 4 to verify the results of the DAG analysis. To sufficiently analyze the results, we also included the longitudinal data (at baseline and week 4) to examine the association found in the DAG analysis. On the one hand, among the variables found in the DAG branch, the levels of the symptoms at baseline were stratified according to the levels of their median, and the two baseline groups' levels of symptoms at week 4 of the two baseline groups at week 4 were compared using the Wilcoxon rank-sum test. On the other hand, the Spearman's correlation was used to analyze the correlation between the change values of any two items that were supposed to have a correlation, as well as the relationship between the baseline level of symptoms and the level of subsequent symptoms at week 4. The curves were fitted through scatter plots.

## Results

### Sample characteristics

Of the 1385 subjects, 31.55% were male. The mean age was 34.21 ± 12.31 years. The majority (58.70%) have a normal and healthy weight. The mean duration of illness was 34.05 ± 51.04 months and 14.15% (*n* = 196) had a family history of mental illness. Data relating to demographics and the clinical characteristics of the study population are summarized in Table 1. The mean scores for the HAMD-17 items, SDS items, and Q-LES-Q-SF total scores are reported in Table 2.

**Table 1.** Basic information *n* (%) or (mean ± s.d.)

Variables	Total	Male	Female
Gender (male:female)	437 (31.55):948 (68.45)		
Age, years	34.21 ± 12.31	34.61 (12.02)	34.02 (12.44)
BMI			
Normal or healthy weight	813 (58.70)	220 (50.34%)	593 (62.55%)
Overweight or obese	369 (26.64)	183 (41.88%)	186 (19.62%)
Underweight	203 (14.66)	34 (7.78%)	169 (17.83%)
Educational level			
Primary school	62 (4.48)	9 (2.06%)	53 (5.60%)
Middle school	197 (14.24)	63 (14.45%)	134 (14.15%)
High school	268 (19.38)	86 (19.72%)	182 (19.22%)
College graduate	706 (51.05)	230 (52.75%)	476 (50.26%)
Postgraduate	150 (10.85)	48 (11.01%)	102 (10.77%)
Employments status			
Employed	755 (54.67)	277 (63.53%)	478 (50.58%)
Unemployed	362 (26.21)	86 (19.72%)	276 (29.21%)
Students	264 (19.12)	73 (16.74%)	191 (20.21%)
Marital status			
Single or divorced	651 (47.11)	202 (46.22%)	449 (47.51%)
Married	731 (52.89)	235 (53.78%)	496 (52.49%)
Clinical feature			
First episode (yes:no)	821 (59.36):562 (40.64)	386 (68.68):176 (31.32)	560 (68.21):261 (31.79)
Duration of illness (months)	34.05 ± 51.04	33.87 (50.54)	34.14 (51.29)
Age of onset	33.41 ± 12.43	33.92 (12.16)	33.18 (12.56)
Duration of current episode (months)	8.31 ± 17.52	8.24 (17.66)	8.34 (17.46)
Family history of mental illness (yes:no)	196 (14.15):1189 (85.85)	72 (16.48):365 (83.52)	124 (13.08):824 (86.92)

Educational level of two patients was missing.

Employments status of four patients was missing.

Marital status of three patients was missing.

First episode information of two patients was missing.

Duration of illness (months) and age of onset of 20 patients were missing.

Duration of current episode (months) of 18 patients was missing.

**Table 2.** The mean scores for the HAMD-17, SDS, and Q-LES-Q-SF (mean ± s.d.)

Variables	Total	Male	Female
HAMD-17 scale			
Depressed mood	2.78 ± 0.80	2.71 (0.80)	2.80 (0.79)
Feelings of guilt	1.25 ± 0.84	1.25 (0.85)	1.25 (0.84)
Suicide	0.97 ± 0.85	0.95 (0.94)	0.98 (0.80)
Insomnia early	1.26 ± 0.82	1.27 (0.83)	1.26 (0.82)
Insomnia middle	1.16 ± 0.74	1.15 (0.77)	1.16 (0.72)
Insomnia late	1.12 ± 0.83	1.15 (0.82)	1.11 (0.84)
Work and activities	2.47 ± 0.84	2.54 (0.87)	2.44 (0.83)
Retardation	0.90 ± 0.68	0.92 (0.64)	0.90 (0.70)
Agitation	0.99 ± 0.79	1.08 (0.81)	0.95 (0.78)
Anxiety psychological	2.02 ± 0.87	2.04 (0.86)	2.01 (0.87)
Anxiety somatic	1.78 ± 0.96	1.72 (0.97)	1.81 (0.95)
Somatic symptoms gastrointestinal	0.92 ± 0.67	0.92 (0.66)	0.93 (0.67)
Somatic symptoms general	1.26 ± 0.66	1.25 (0.66)	1.26 (0.66)
Genital symptoms	0.70 ± 0.75	0.78 (0.76)	0.66 (0.74)
Hypochondriasis	0.95 ± 0.92	0.99 (0.95)	0.93 (0.91)
Loss of weight	0.61 ± 0.81	0.65 (0.83)	0.59 (0.80)
Insight	0.41 ± 0.54	0.45 (0.58)	0.39 (0.52)
Total score of HAMD17	21.54 ± 5.05	21.80 (5.20)	21.42 (4.97)
Total score of Q-LES-Q-SF	34.75 ± 6.93	34.68 (7.02)	34.78 (6.89)
SDS scale			
Work or school	5.66 ± 2.92	5.85 (2.90)	5.57 (2.93)
Social life	5.56 ± 2.64	5.65 (2.64)	5.52 (2.64)
Family life	5.14 ± 2.79	5.01 (2.83)	5.20 (2.77)
Total score of SDS	16.36 ± 7.14	16.51 (7.17)	16.30 (7.13)

Note: HAMD-17 is the 17-item Hamilton Rating Scale for Depression, SDS is the Sheehan Disability Scale, Q-LES-Q-SF is the Quality of Life Enjoyment and Satisfaction Questionnaire – Short Form.

### Undirected network structure (graphical LASSO)

**Fig. 1.**
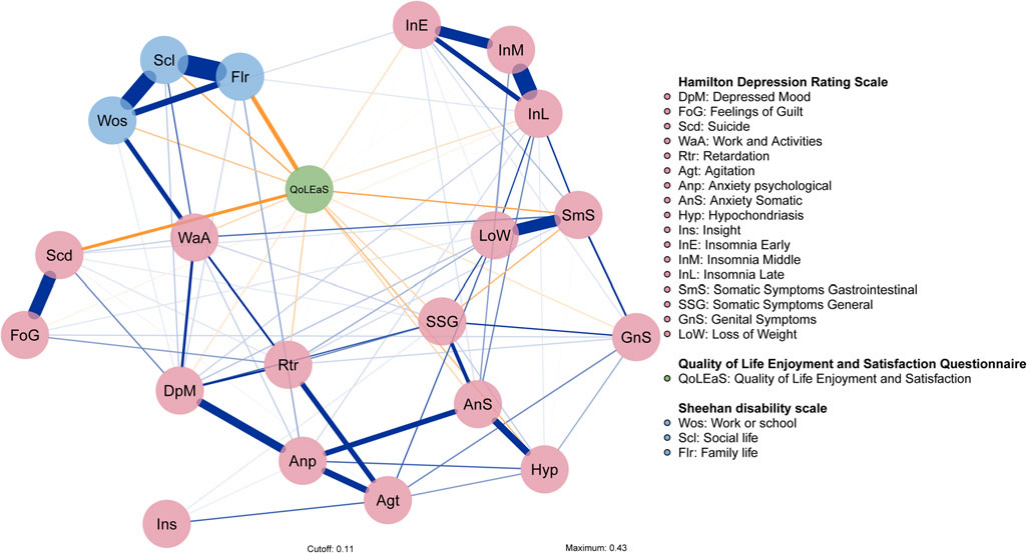
Undirected networks of depressive symptoms, functional disabilities, and QOL in patients with MDD. Each edge corresponds to a partial correlation (positive in blue, negative in orange, maximum magnitude = 0.43) between two items; the thickness corresponds to the absolute magnitude of the correlation. The colors of the nodes correspond to detected communities in the network: HAMD-17 (pink), SDS (blue), and Q-LES-Q-SF total score (green). Item label abbreviations are defined on the right side of figure.

The network for depressive symptoms, functional impairment, and QOL contained 21 nodes (Fig. 1). Most of the edges among depressive symptoms and functional impairment represent positive correlations; however, all links with life satisfaction were negative. The strongest edges were observed between the life satisfaction and family life functional disability nodes, as well as the suicidal ideation node. Nodes within the domain of functional impairment were generally highly interconnected and clustered closely together. Within the depressive symptoms, strong links were found between suicidal ideation and feelings of guilt, depressed mood, and psychological anxiety, as well as early, middle, and late insomnia.

**Fig. 2.**
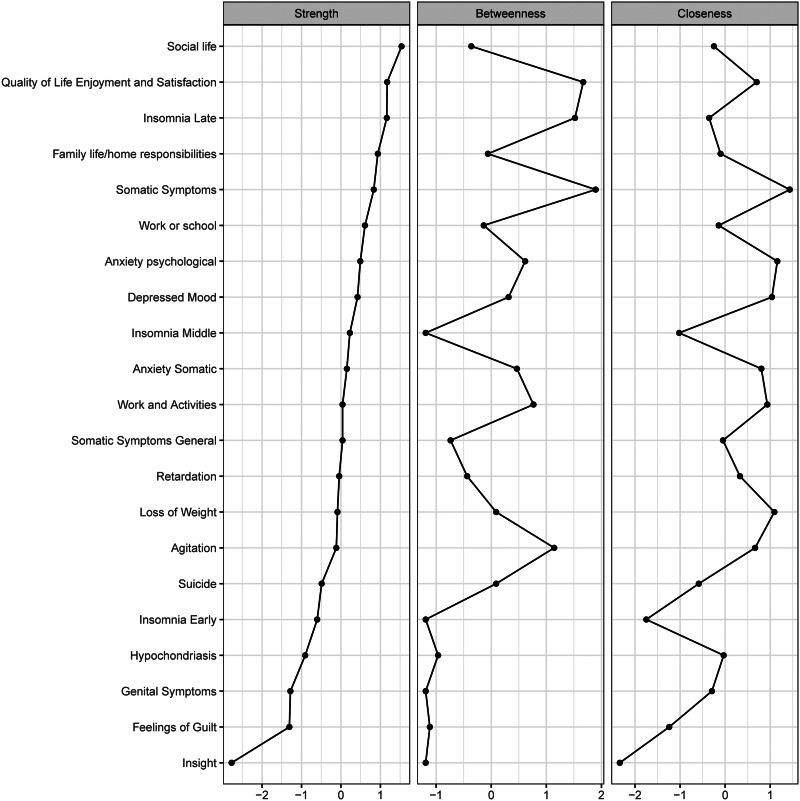
Node centrality metrics of the network. The left, middle, and right panels show the strength, betweenness, and closeness estimates for each node of the network, respectively.

Three centrality metrics (strength, betweenness, and closeness) of the graphical LASSO are presented in Fig. 2. A node with a higher index of centrality is more central in the network, and this implies close linkage to other symptoms in the network (Opsahl et al., 2010). The five symptoms exhibiting the highest strength centrality were social life dysfunction, QOL, late insomnia, family life responsibilities, and somatic symptoms. Somatic symptoms and QOL emerged as the most central nodes in the network for betweenness, indicating that these two symptoms often transmitted information through the network (McElroy, Shevlin, Murphy, & McBride, 2018). Somatic symptoms and psychological anxiety had the highest centrality for closeness, indicating that these are key nodes that are more likely to affect other symptoms in a rapid manner (Bringmann, Lemmens, Huibers, Borsboom, & Tuerlinckx, 2015).

**Fig. 3.**
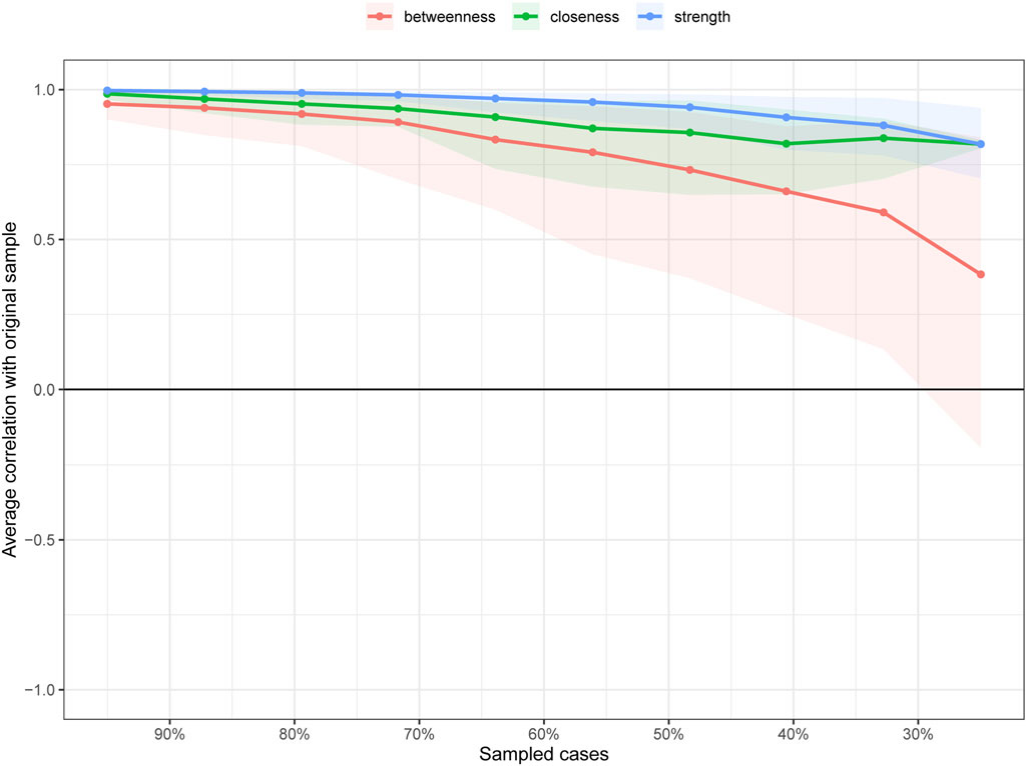
Stability of the centrality indices: point estimates and corresponding 95% CIs. This was determined by average correlations between the centrality indices of networks sampled with patients dropped and the original sample. Lines indicate the means and areas indicate the range from the 2.5th quantile to the 97.5th quantile.

Data acquired through the case-drop procedure suggested a robust estimation of centrality (Fig. 3). The correlations between the measures of centrality using the full sample and a subset of 30% of the data were higher than 0.25. This indicates that, even when 70% of the sample were randomly removed, the network structure remained highly stable. The network also appeared quite stable across bootstrapped difference tests for edge weights and node strength (online Supplementary Figs S1 and S2). The result of bootstrapped 95% confidence intervals for the edge weights is shown in online Supplementary materials (Fig. S3).

The results relating to gender difference in the network indicated that the global network strength was larger in the female network than in the male network (*S* = 2.202, *p* = 0.0278), although this was not accompanied by differences in the network structure (*M* = 0.149, *p* = 0.1008). Depressed mood symptom plays a more central role in the female network, while in the male network this role is played by somatic symptoms. These results are shown in the online Supplementary material (Figs S4–S6).

### Bayesian network

**Fig. 4.**
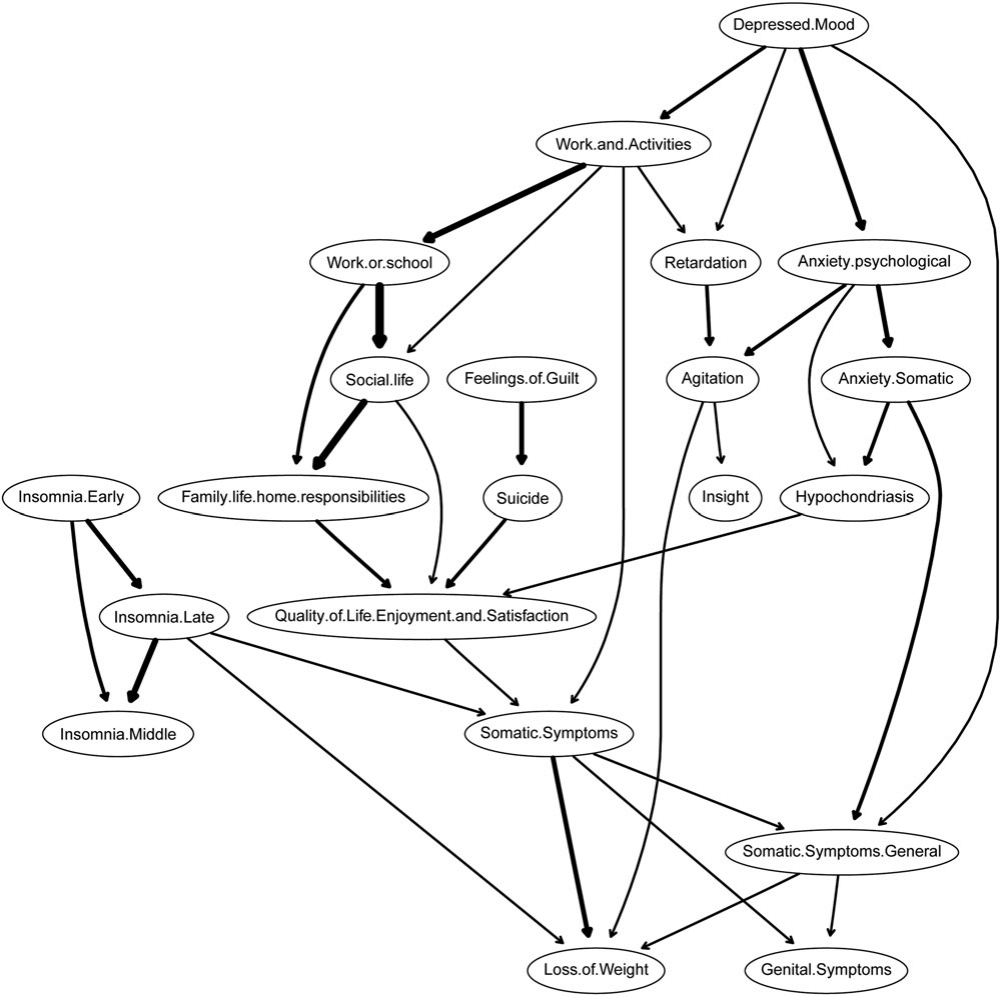
Directed acyclic graph (DAG) of depressive symptoms, functional disabilities, and QOL in patients with MDD. Arrows indicate the direction of the assumed causal relationships. Edge thickness indicates confidence that the predicted direction of edge points in the direction displayed in the graph.

Figure 4 depicts the DAG arising from the Bayesian network analysis and illustrates connections between depressive symptoms, functional disabilities, and QOL in patients with MDD. Edge thickness indicates the importance of the connections in terms of network structure; the position of different items is indicative of predictive priority over other items. Depressed mood was positioned at the highest level in the model, suggesting its causal priority. Depressed mood predicted the activation of functional impairment, including disability related to work, activities, social life, and family life, thus activating the QOL. In another important branch, the depressed mood node triggered anxiety including psychological anxiety, somatic anxiety, and general somatic symptoms. As evident in the regularized partial correlation network, a feeling of guilt directly influences suicidal ideation (with a strong association), thus influencing the QOL. While the DAG estimated several branches of activation, some nodes (e.g. QOL and somatic symptom) appeared to act as ‘bridges’ between these pathways. In total, 1025 patients with MDD (74.0% of the total sample) were followed at week 4 (HAMD-17 total score = 11.07 ± 6.31). Generally, the DAG of the week 4 follow-up data was consistent with the baseline result. The three impact pathways identified by the baseline DAG were validated by the 4-week data, namely (first branch: depressed mood – disrupted work and activities – disrupted social life – disrupted family life – QOL; second branch: depressed mood – psychological anxiety – somatic anxiety – general somatic symptoms; third branch: feeling of guilt – suicidal ideation – QOL). These results are shown in the online Supplementary material (Fig. S8).

**Fig. 5.**
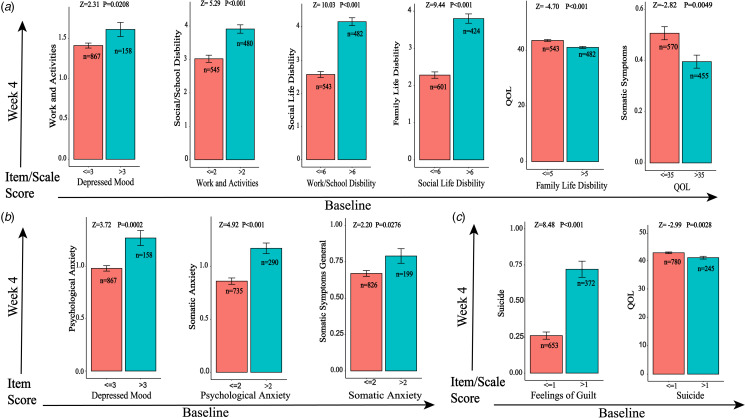
Longitudinal data (baseline and week 4) used to examine the association found in DAG analysis. *Note:* (a) Branch 1: depressed mood – work and activity – work or school disability – social life disability – family life disability – quality of life – somatic symptoms; (b) Branch 2: depressed mood – psychological anxiety – somatic anxiety – somatic symptoms general; (c) Branch 3: feelings of guilt – suicide – quality of life. The levels of the symptoms at baseline were stratified according to the levels of their median. The groups with lower baseline item/scale score are depicted in red, and the groups with higher baseline score are in teal.

The result of the analysis using the longitudinal data indicated that compared with the group with a lower baseline level of depressive symptoms in the DAG branch, the group with a higher baseline level has a higher level of subsequent symptoms at week 4, *p* < 0.05 (Fig. 5). And this between-group difference was not found between the variables with no connections indicated in the DAG (e.g. the suicide item and the insomnia middle item; the insight item and the feelings of guilt item), *p* > 0.05 (online Supplementary Fig. S9). A significant correlation was found between the baseline level of symptoms and the level of subsequent symptoms at week 4 (e.g. the depressed mood item at baseline was positively associated with the work and activity item at week 4). The curves were also fitted through scatter plots (online Supplementary Fig. S10). The correlation between the change values (from baseline to week 4) of any two items in the DAG branch that were supposed to have a correlation was significant. For example, the reduction of the score of the work and the activity items was positively associated with the reduction of the score of the depressed mood item (*r* = 0.25, *p* < 0.001), the reduction of psychological anxiety was positively associated with the reduction of the score of the depressed mood item (*r* = 0.37, *p* < 0.001), and the reduction of the score of the suicide item was positively associated with the reduction of the score of the feelings of guilt item (*r* = 0.26, *p* < 0.001). The curves were also fitted through scatter plots (online Supplementary Fig. S11).

## Discussion

To the best of our knowledge, this is the first study to examine the associations between depressive symptoms, functional impairment, and QOL in major depressive patients using both undirected and Bayesian network analyses. The sample is representative of the general population, to an extent. On the one hand, the male-to-female ratio in our study was close to 1:2. It is in line with the gender difference in the prevalence of depression that women are twice as likely to experience depression compared with men, and this finding has been replicated across many cultures as one of the most robust findings in psychiatric epidemiology (Bone, Lewis, & Lewis, 2020; Salk, Hyde, & Abramson, 2017). On the other hand, the participants of this study had various demographic and clinical characteristics, e.g. education levels, employments status, marital status, episodes, and duration of illness. The results clearly indicated that functional disability was an important mediator between depressive symptoms and QOL. Depressed mood played a predominant role in activating both anxiety symptom and functional impairment. The factors most directly related to QOL were family functionality and suicidal symptoms.

Our results indicated that some depressive symptoms (e.g. suicidal ideation and hypochondriasis) were directly associated with QOL, while other depressive symptoms (e.g. depressed mood and a loss of interest in activity, hobbies, or work) showed an indirect association with QOL mediated by impaired function. These results are examined using longitudinal data available and are consistent with the results of most previous studies that attempted to identify the relationship between depressive symptoms, family function, and QOL (Akosile et al., 2018; Alessandrini et al., 2016; Gardsjord et al., 2018). The indirect path suggests that the depressive symptoms' impact on function can cause an alteration of QOL, not necessarily the symptoms themselves. Alessandrini et al. also revealed a highly significant path between depression and QOL, as well as a minor significant indirect path between depression and QOL mediated by function, using a structural equation modeling approach (Alessandrini et al., 2016). Conversely, some previous studies showed that depression is an intermediary factor for the relationship between function and QOL (Chan, Pan, Xu, & Yeung, 2021; Clements, Frazier, Moser, Lennie, & Chung, 2020; Lu, Yuan, Lin, Zhou, & Pan, 2017). The reasons for this inconsistency were likely different study populations and measurement models.

As one of core symptoms defined by DSM-5 to diagnose MDD (APA., 2013), depressed mood was located at the top of the DAG. Therefore, depressed mood was estimated to have greater priority and to contribute to the rapid activation of interrelated symptoms (Armour, Fried, Deserno, Tsai, & Pietrzak, 2017). It should be noted that, in this result, depressed mood induced functional impairment through the loss of interest in activity, hobbies, or work. These results underline the potential clinical importance of the core criterion, and support a previous study reporting that the core symptoms of DSM were of the highest impact in terms of the impairment of psychosocial functioning (Fried & Nesse, 2014). Moreover, depressed mood also triggered the activation of anxiety symptoms. Anxiety was proposed to be reclassified within depressive disorders as a continuum from depression to anxiety (Park & Kim, 2020). These conditions overlap substantially and are organized within a larger psychopathological network (Cramer, Waldorp, van der Maas, & Borsboom, 2010; Fried & Nesse, 2015; Goekoop & Goekoop, 2014). This means that once a few specific symptoms have been activated, these activated signals can spread from symptoms of depression to those of anxiety *via* highly central symptoms (Fried, Epskamp, Nesse, Tuerlinckx, & Borsboom, 2016). Some findings show that the symptoms of depression are no more central than anxiety symptoms for patients with MDD (Park & Kim, 2020). For example, the results of the STAR*D study suggested that DSM symptoms (e.g. sad mood) and non-DSM symptoms (e.g. anxiety) were among the most central symptoms, while DSM criteria were no more central than non-DSM criteria (Fried et al., 2016). However, our results showed that ‘depressed mood’ was still the key symptom that contributes to the rapid activation of interrelated symptoms and dysfunction. So, we emphasized the importance of ‘depressed mood’ as the therapeutic target for early intervention. A possible reason for the difference between the results of our study and that of STAR*D is that the network analysis in STAR*D focused purely on the comparison among depressive symptoms, whereas our study had some expansion on the dimensions of social functioning and QOL.

Our results indicated that family functionality and suicidal symptoms were the factors that were most directly related to QOL. Both undirected and directed analyses identified a connection between suicidal ideation and a reduced QOL and the most likely direction was from suicidal ideation to QOL, rather than *vice versa*. This was in line with other studies finding that suicidal ideation was associated with QOL (Almeida et al., 2021; Beşirli, Alptekin, Kaymak, & Özer, 2020; van Spijker et al., 2020) and suicidal thinking may reduce QOL (Hidalgo-Rasmussen & Martín, 2015; Kim et al., 2019; van Spijker, van Straten, Kerkhof, Hoeymans, & Smit, 2011). It should be noted that there are also some previous studies indicating that a low QOL could be a risk factor for suicidal ideation (Fairweather-Schmidt, Batterham, Butterworth, & Nada-Raja, 2016; Faure et al., 2018; Sinclair, Hawton, & Gray, 2010; van Spijker et al., 2020). For instance, in a prospective study based on a Finnish twin cohort, QOL was found to exert a long-term effect on the risk of suicide throughout a 20-year follow-up period (Koivumaa-Honkanen et al., 2001). Additionally, a study found that functional impairment in family life/home responsibilities would lead to a direct reduction in QOL. One explanation could be that family relationships are usually considered the major source of emotional comforts which exert a significant influence on the QOL (Lu et al., 2017).

Centrality analysis indicated that social life dysfunction exhibited the highest strength centrality. The potential explanation for this finding is that social life dysfunction is often manifested as hyper-sensitivity to social rejection and impaired social perception (Kupferberg, Bicks, & Hasler, 2016). These conditions predict higher rates of internal life stressors (Liu, Kraines, Massing-Schaffer, & Alloy, 2014) and subsequent depressive symptoms (Liu et al., 2014). It should be admitted that betweenness and closeness centrality seem to be especially poorly suited to most psychological networks, as they come with strong assumptions that may not hold. However, many alternative measures that were introduced over a substantial period of time also have these problems (Bringmann et al., 2019). Thus, strength, betweenness, and closeness are still the most common centrality indices used in psychological network research (Ye et al., 2021).

About the networks developed in terms of gender, we found significant differences in the network global strength. Symptom networks of female patients were more strongly connected, as global strength was higher in the female group than in the male group, and this result is consistent with previous findings (Jin et al., 2022; Steen, van Borkulo, & van Loo, 2021). This result supports the notion that the cluster of dynamic mechanisms of depressive symptoms in women is more complex compared with men (Cramer et al., 2016), which may be related to a greater vulnerability to depression and a less positive prognosis (van Borkulo et al., 2015). On the other hand, our results indicated that symptoms central to the network were similar in general between the sexes. While depressed mood has a greater presence in women, somatic symptoms are more central in men. This finding supports the Gender Responding Framework (Castellanos, Ausin, Bestea, Gonzalez-Sanguino, & Munoz, 2020; Price, Gregg, Smith, & Fiske, 2018; Rodgers et al., 2014), which indicated that men tend to present more externalized symptoms, and women tend to present more internalized symptoms. These differences can be critical for providing important additional information about the relationships among depression symptoms in males and females, as well as making a more complete evaluation of MDD.

This study was strengthened by a large, multi-site sample of individuals suffering from MDD and the use of network models that are appropriate for describing complex dynamic systems (Nelson, McGorry, Wichers, Wigman, & Hartmann, 2017). However, our study has some limitations that need to be mentioned. First, even though the DAG provides preliminary clues to both the strength and direction of the possible connections among the variables under analysis, caution is still required as causal inference is only fully defensible when all assumptions are completely met (Moffa et al., 2017). Longitudinal studies focusing on disease development are needed for further validation (Bringmann et al., 2013). Second, although a wide range of clinical dimensions were assessed, these network structures must be explained under the assumption of the absence of unmeasured confounders or selection variables. These variables may include shame, self-esteem, or cognitive biases. The inclusion of those variables in future network-based studies may enrich the research of MDD. Third, the study excluded patients with suicidal ideation item score >2 (as high risk for suicide), which may impact the relationship of suicidal ideation with other depressive symptoms, QOL, and functioning.

Despite these limitations, to our knowledge, this study was the first to elucidate the associations between depressive symptoms, functional impairment, and QOL in patients with MDD at the level of individual symptoms. We found that functional disability mediated the relationship between depressive symptoms and QOL. Depressed mood played the predominant role in activating both anxiety symptoms and functional impairment. Family functionality and suicidal symptoms were directly related to QOL. The study has important guiding value for the further understanding of MDD and the adjustment of treatment strategies for patients with this disease.

## Supplementary material

For supplementary material accompanying this paper visit https://doi.org/10.1017/S0033291722003385. click here to view supplementary material
