# Supplementary material for: The associations between depressive symptoms, functional impairment, and quality of life, in patients with major depression: undirected and Bayesian network analyses
Source: Psychol Med. 2022 Nov 9;53(14):6446–58. doi: 10.1017/S0033291722003385 (PMC10600944; doi:10.1017/S0033291722003385)
Supplement: Supplementary file 1 [file S0033291722003385sup001.docx]

Supplementary materials for manuscript

The associations between depressive symptoms, functional impairment, and quality of life, in patients with major depression: undirected and Bayesian network analyses

Jia Zhou ^1, 2 #^, Jingjing Zhou^1, 2, #^, Lei Feng^1, 2,^ Yuan Feng^1, 2,^ Le Xiao^1, 2,^ Xu Chen^1, 2,^ Jian Yang^1, 2, *^ Gang Wang^1, 2, *^

1 The National Clinical Research Center for Mental Disorders & Beijing Key Laboratory of Mental Disorders, Beijing Anding Hospital, Capital Medical University, Beijing, China.

2 Advanced Innovation Center for Human Brain Protection, Capital Medical University, Beijing, China, Beijing, China.

# These authors contributed equally to this work.

*Corresponding authors

Contents:

1. Table S1: Assigned label and wording of the HAMD-17, SDS and Q-LES-Q-SF.

2. Robustness analyses for overall data

2.1. Figure S1: Strength differences

2.2. Figure S2: Edge weight differences

2.3 Figure S3 Bootstrapped confidence intervals of all edge weights

3. Male and female network analysis

3.1 Figure S4: Male network structure

3.2 Figure S5: Female network structure

3.3 Figure S6: Node centrality metrics of female and male network structure

4. Figure S7: Network structure with items of the Q-LES-Q-SF included

5. Figure S8: Directed acyclic graph (DAG) of depressive symptoms, functional disabilities and QOL in MDD patients at week 4.

6. Longitudinal data was included to examine the found of DAG

6.1 Figure S9: Between-group difference of variables that were not indicated in the DAG

6.2 Figure S10. The relationship between baseline level of symptoms and level of subsequent symptoms at week 4

6.3 Figure S11. The correlation between the change values of any two items that were supposed to have a correlation

**1. Table S1. Assigned label and wording of the HAMD-17, SDS and Q-LES-Q-SF.**

| *Table S1. Assigned label and wording of HAMD-17, SDS and Q-LES-Q-SF. Scale* | *Items* | *Wording* | *Label* |
| --- | --- | --- | --- |
| ***HAMD-17*** | *Depressed Mood* | *Sadness‚ hopeless‚ helpless‚ worthless.* | *DpM* |
|  | *Feelings of Guilt* | *Self-reproach,* *ideas of guilt or rumination,* *delusions of guilt,* *hears accusatory or denunciatory voices and/or experiences threatening visual hallucinations.* | *FoG* |
|  | *Suicide* | *Feels life is not worth living, wishes he were dead or any thoughts of possible death to self, suicidal ideas or gesture, attempts at suicide.* | *Scd* |
|  | *Insomnia Early* | *Complains of difficulty falling asleep.* | *InE* |
|  | *Insomnia Middle* | *Patient complains of being restless and disturbed during the night, waking during the night.* | *InM* |
|  | *Insomnia Late* | *Waking in early hours of the morning.* | *InL* |
|  | *Work and Activities* | *Incapacity‚ fatigue or weakness related to activities‚ work or hobbies.* | *WaA* |
|  | *Retardation* | *Slowness of thought and speech; impaired ability to concentrate; decreased.* | *Rtr:* |
|  | *Agitation* | *Moving about‚ can’t sit still, hand wringing‚ nail biting‚ hair-pulling‚ biting of lips.* | *Agt* |
|  | *Anxiety psychological* | *Subjective tension and irritability, worrying about minor matters, apprehensive attitude apparent in face or speech, fears expressed.* | *Anp* |
|  | *Anxiety Somatic* | *Physiological concomitants of anxiety (i.e.‚ effects of autonomic over activity‚ “butterflies‚” indigestion‚ stomach cramps‚ belching‚ diarrhea‚ palpitations‚ hyperventilation‚ paresthesia‚ sweating‚ flushing‚ tremor‚ headache‚ urinary frequency). Avoid asking about possible medication side effects (i.e.‚ dry mouth‚ constipation).* | *AnS* |
|  | *Somatic Symptoms (gastrointestinal)* | *Loss of appetite, difficulty eating.* | *SmS* |
|  | *Somatic Symptoms General* | *Heaviness in limbs‚ back or head. Backaches‚ headache or muscle aches. Loss of energy and fatigability.* | *SSG* |
|  | *Genital Symptoms* | *Symptoms such as loss of libido; impaired sexual performance; menstrual disturbances.* | *GnS* |
|  | *Hypochondriasis* | *Self-absorption (bodily), preoccupation with health, frequent complaints‚ requests for help‚ hypochondriacal delusions.* | *SSG* |
|  | *Loss of Weight* | *Weight loss associated with present illness.* | *LoW* |
|  | *Insight* | *Acknowledges being depressed and ill.* | *Ins* |
| *SDS* | *Work or school* | *The symptoms have disrupted your work/school work* | *Wos* |
|  | *Social life* | *The symptoms have disrupted your social life/leisure activities* | *Scl* |
|  | *Family life* | *The symptoms have disrupted your family life/home responsibilities* | *Flr* |
| *Q-LES-Q-SF* | *Quality of Life Enjoyment and Satisfaction* | *Quality of Life Enjoyment and Satisfaction* | *QoLEaS* |

**2. Robustness analyses for overall data**

All robustness analyses were conducted using the bootnet package for R. An accessible tutorial paper for the procedures has been published (Epskamp and Fried, 2018).


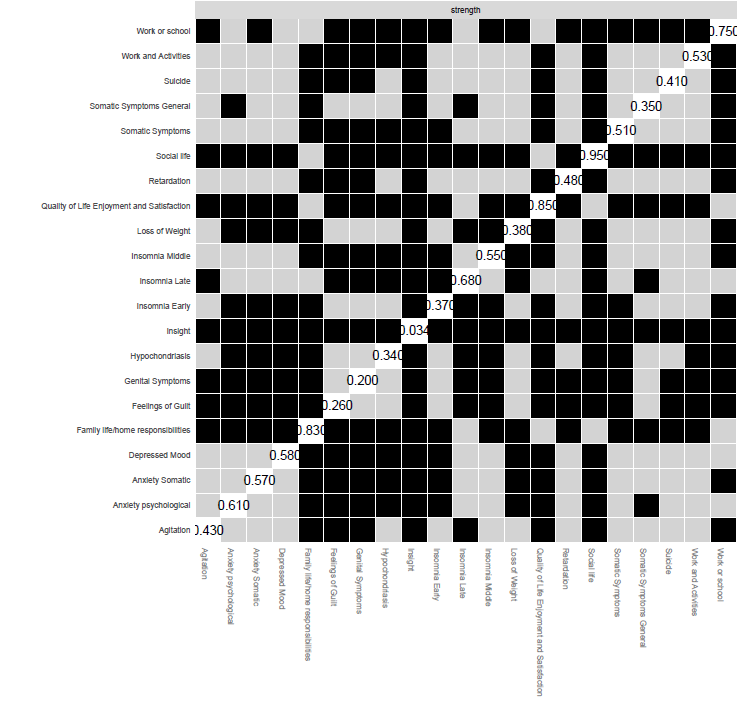


**Figure S1.** Strength differences at baseline

*Note: Nodes that differ from each other in strength, i.e. the combination of all edge weights connecting to that node. Black boxes represent significant differences, and strength values are in the diagonal.*


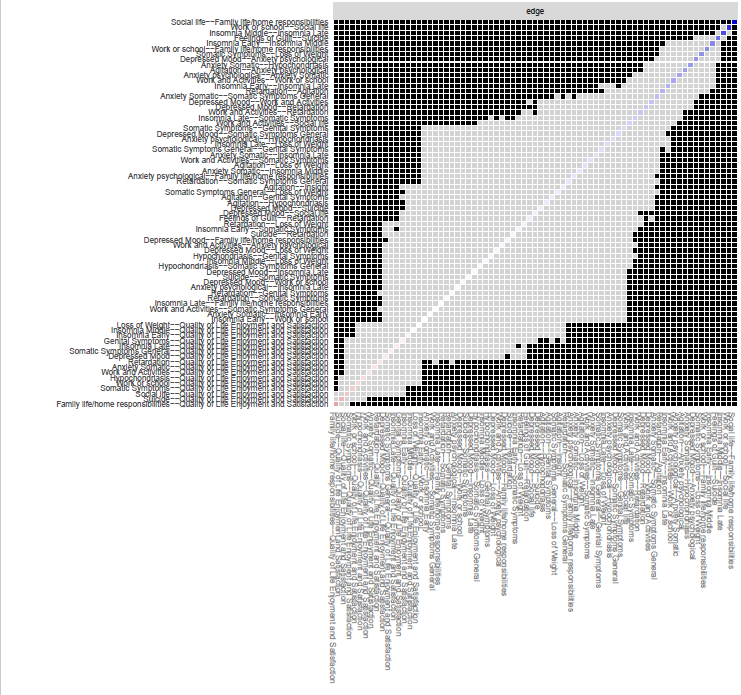


**Figure S2.** Edge weight differences at baseline. Edges that differ significantly from each other are depicted by black boxes.


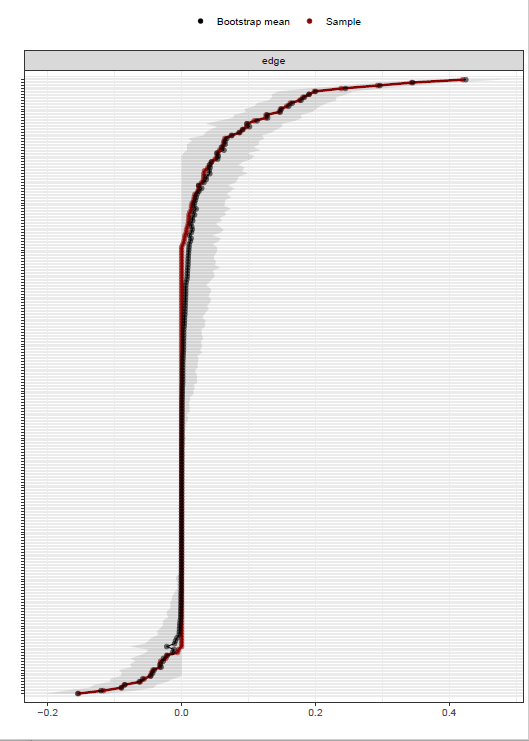


**Figure S3.** Bootstrapped confidence intervals of all edge weights *Note: Red line indicates sample means, black line indicates bootstrapped means and grey area indicates 95% confidence intervals for individual edges. Narrower intervals suggest more precise estimates and overlap of intervals suggests statistically non-significant differences between edges.*

**3. Male and female network analysis**


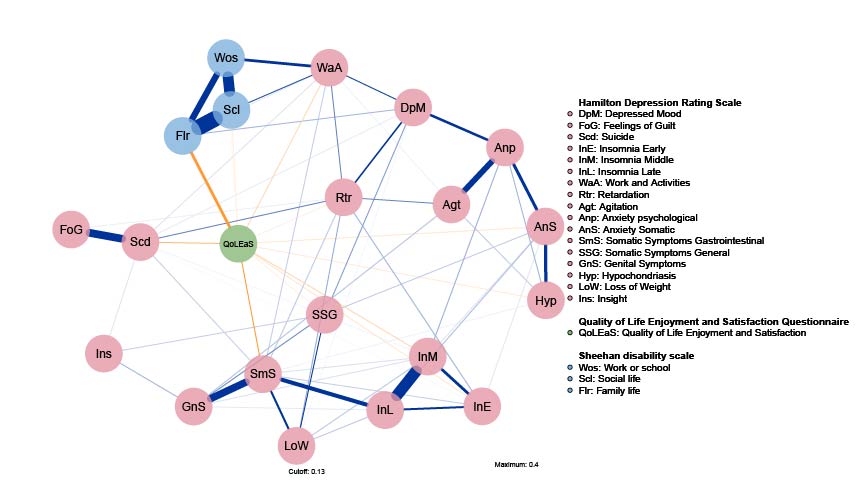


**Figure S4.** Male network structure.

*Note: Each edge corresponds to a partial correlation (positive in blue, negative in orange) between 2 items, and its thickness corresponds to the absolute magnitude of the correlation. Colors of the nodes correspond to detected communities in the network: HAMD-17 (pink), SDS (blue), and Q-LES-Q-SF total score (green). Item label abbreviations are defined on the right side of the figure.*


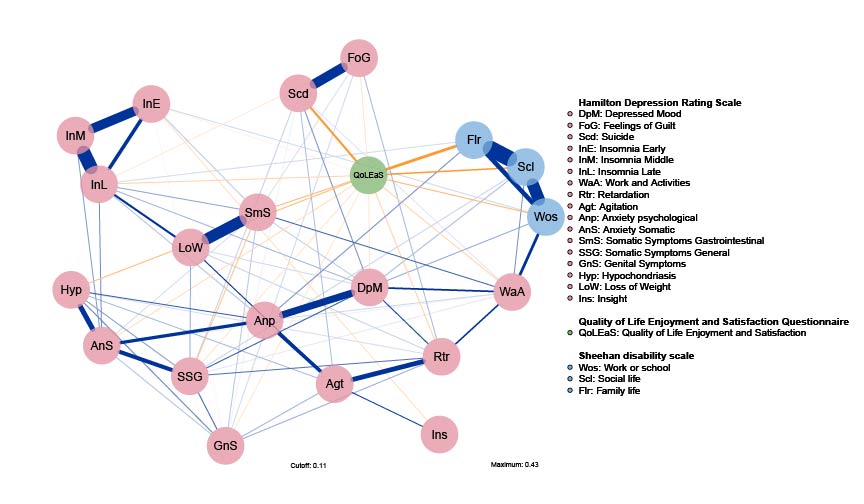


**Figure S5.** Female network structure

*Note: Each edge corresponds to a partial correlation (positive in blue, negative in orange) between 2 items, and its thickness corresponds to the absolute magnitude of the correlation. Colors of the nodes correspond to detected communities in the network: HAMD-17 (pink), SDS (blue), and Q-LES-Q-SF total score (green). Item label abbreviations are defined on the right side of the figure.*


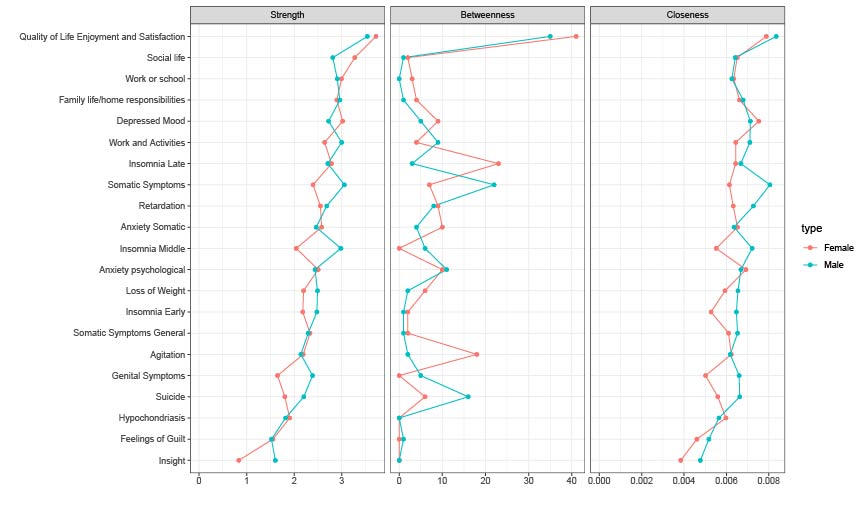


**Figure S6.** Node centrality metrics of female and male network structure.

*Note: Node centrality metrics of the network. Left, middle, and right panel shows the strength, betweenness, and closeness estimates for each node of the network, respectively.*

**4. Network structure with items of the Q-LES-Q-SF included**


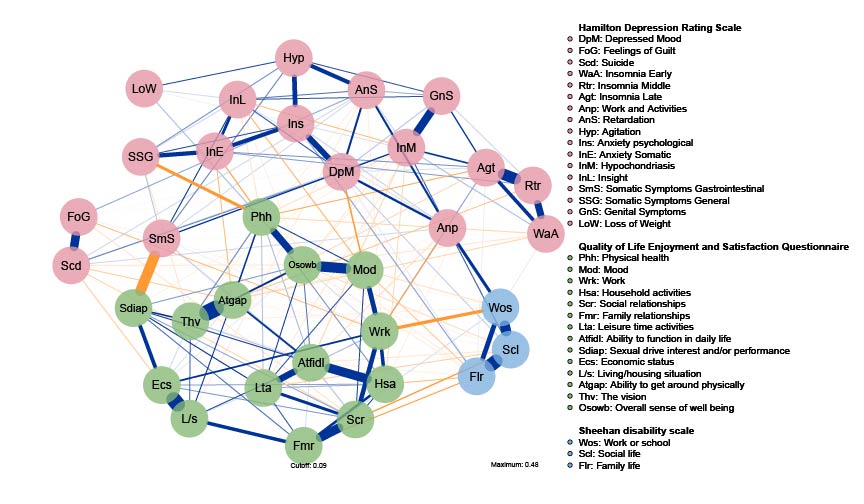


**Figure S7**. Undirected networks of depressive symptoms, functional disabilities and QOL in MDD patients

*Note: Items of Q-LES-Q-SF were included. Each edge corresponds to a partial correlation (positive in blue, negative in orange) between 2 items, and its thickness corresponds to the absolute magnitude of the correlation. Colors of the nodes correspond to detected communities in the network: HAMD-17 (pink), SDS (blue), and Q-LES-Q-SF total score (green). Item label abbreviations are defined on the right side of the figure.*

**5. Directed Acyclic Graph (DAG) of depressive symptoms, functional disabilities and QOL in MDD patients at week-4.**


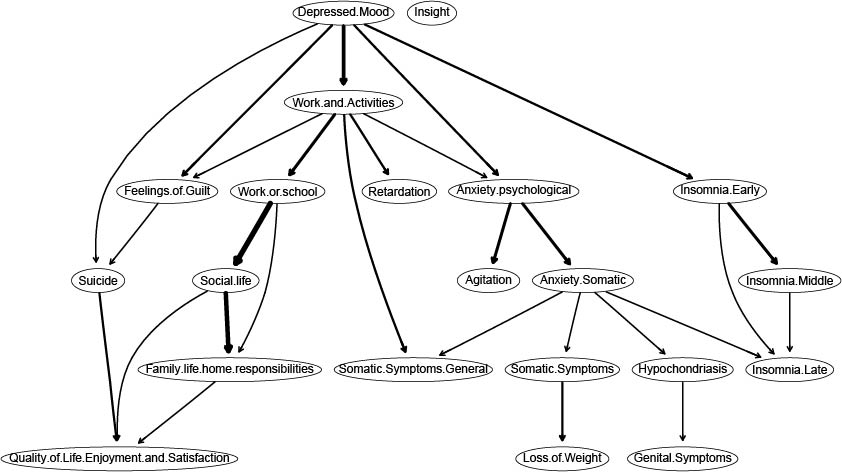


**Figure S8.** Directed Acyclic Graph (DAG) of depressive symptoms, functional disabilities and QOL in MDD patients at week 4.

*Note:* *1025 MDD patients were followed at week 4 (HAMD-17 total score=11.07±6.31).*

*Arrows indicate the direction of the assumed causal relationships. Edge thickness indicates confidence that the predicted direction of an edge points in the direction displayed in the graph.*

**6.** **Longitudinal data was included to examine the found of DAG**


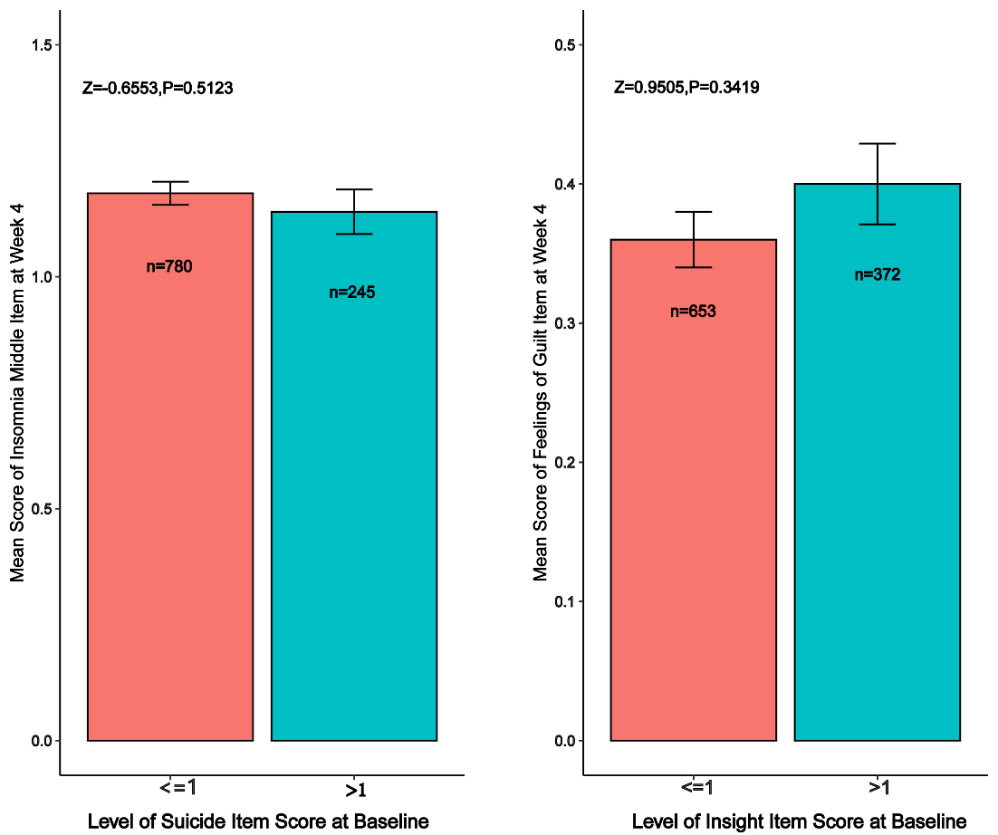


**Figure S9.** Between-group difference of variables that were not indicated in the DAG


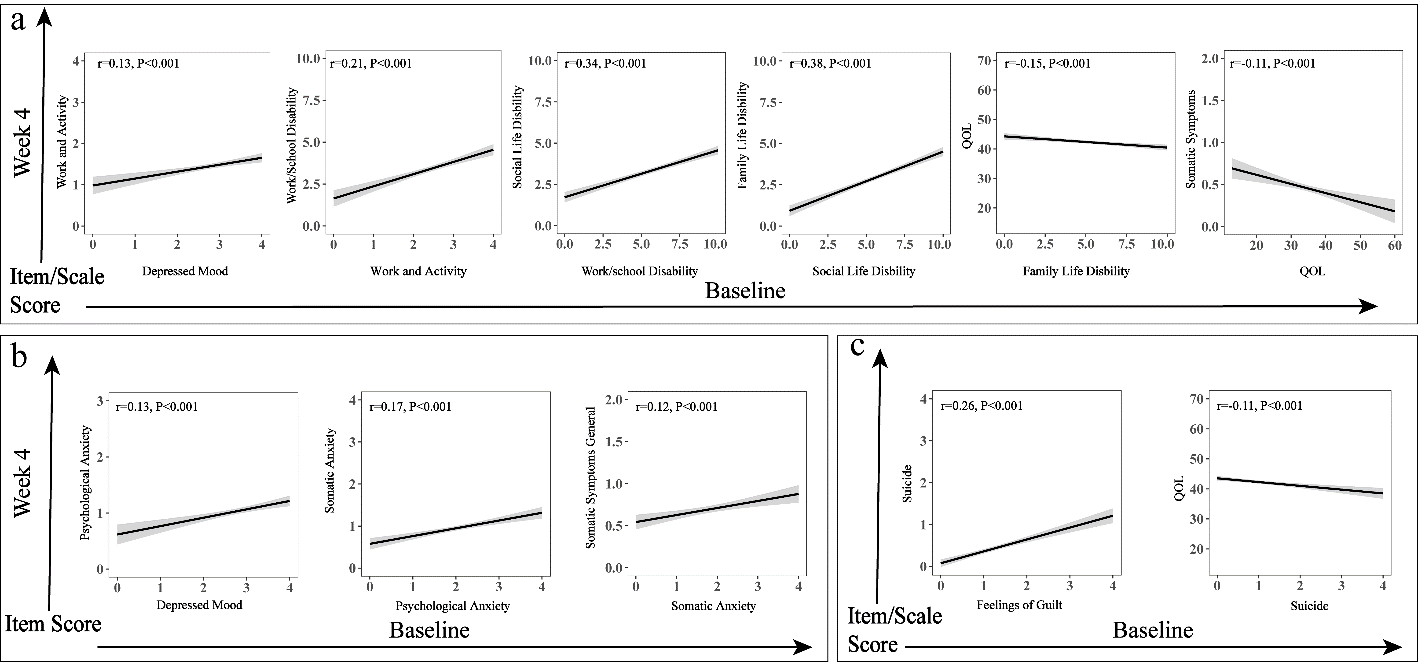


**Figure S10.** The relationship between baseline level of symptoms and level of subsequent symptoms at week 4

*Note: The curves were fitted through scatter plots.*


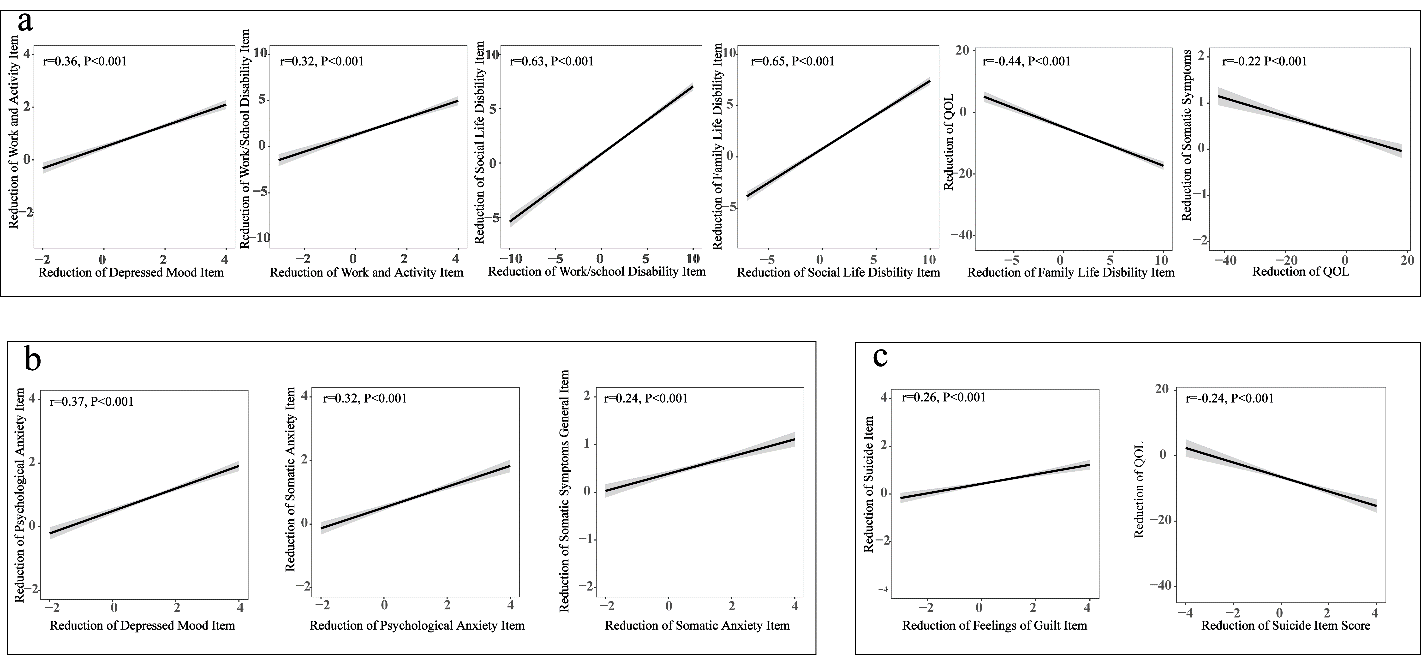


**Figure S11.** The correlation between the change values of any two items that were supposed to have a correlation

*Note: The curves were fitted through scatter plots.*
